# Peptide receptor radionuclide therapy for ectopic Cushing’s syndrome caused by metastatic neuroendocrine neoplasms

**DOI:** 10.1530/EO-24-0013

**Published:** 2024-11-20

**Authors:** Emma Boehm, Terry Hung, Tim Akhurst, Ramin Alipour, Cherie Chiang, Rodney J Hicks, Michael S Hofman, Aravind S Ravi Kumar, Nirupa Sachithanandan, Javad Saghebi, Michael Michael, Grace Kong

**Affiliations:** 1 Department of Molecular Imaging and Therapeutic Nuclear Medicine, Peter MacCallum Cancer Centre, Melbourne, Australia; 2 Department of Internal Medicine, Endocrinology, Peter MacCallum Cancer Centre, Melbourne, Australia; 3 Department of Medicine, St Vincent’s Medical School, The University of Melbourne, Melbourne, Australia; 4 Sir Peter MacCallum Department of Oncology, University of Melbourne, Melbourne, Australia; 5 Department of Medical Oncology, Peter MacCallum Cancer Centre, Melbourne

**Keywords:** ectopic Cushing’s syndrome, neuroendocrine neoplasms, peptide receptor radionuclide therapy, radionuclide therapy, theranostics

## Abstract

**Background:**

Metastatic gastroenteropancreatic neuroendocrine neoplasms (GEPNEN) can cause ectopic Cushing’s syndrome (ECS). ECS is highly morbid and medical therapy is complex and can be ineffective. Patients unsuitable for bilateral adrenalectomy (BA) have dismal outcomes. Peptide receptor radionuclide therapy (PRRT) is a rational option for hormone and disease control in ECS caused by NEN with high somatostatin receptor (SSTR) expression.

**Aim:**

To describe the characteristics and outcomes of patients with ECS treated with PRRT.

**Methods:**

Single-centre, retrospective analysis of imaging, biochemistry and outcomes of seven consecutive patients with ECS caused by metastatic GEPNEN treated with PRRT from 2006 to 2023.

**Results:**

Patients were aged 17–75 (female *n* = 6). The primary site was the pancreas (5/7) and rectum (2/7). Six patients were on medical therapy for ECS at baseline (one had a previous BA). A median of 34.4 GBq of [^177^Lu]Lu-DOTA-octreotate was given. [^90^Y]Y-DOTA-octreotate (one patient) and [^111^In]In-octreotide (one patient) were also used. Five patients had radiosensitising chemotherapy. Five patients had a flare of ECS within 1 week of PRRT cycle 1 (PRRT-C1). Following PRRT-C1, 5/7 patients had complete biochemical resolution of ECS at 1.5–6 months (four ongoing; one recurred after 12 months and had elective BA at 18 months). Best metabolic response on [^18^F]F-FDG PET/CT: Four patients had a complete metabolic response (CMR), and one had a partial metabolic response. PFS was 3–208 months. Two patients progressed at the first follow-up. The longest ECS remission and CMR continues at >17 years.

**Conclusion:**

PRRT can be effective for ECS caused by metastatic SSTR-positive GEPNEN and should be considered in its treatment algorithm.

## Introduction

Ectopic Cushing’s syndrome (ECS) is a rare and deadly consequence of ACTH (and, rarely, CRH) production by neuroendocrine neoplasms (NEN), leading to unregulated adrenal cortisol secretion. ECS can be caused by various NENs including lung (small cell lung cancer or bronchial neuroendocrine tumours (NETs)), thymic NETs, medullary thyroid cancer, gastroenteropancreatic (GEP) NEN, phaeochromocytoma and other rarer sites (Young *et al.* 2020). The mechanism underlying ectopic ACTH hypersecretion across these diverse tissue types has not been clearly elucidated but is hypothesised to include events associated with high-grade disease, including hypomethylation of pro-opiomelanocortin promoter sites (Araki *et al.* 2021) and gene fusions (Agaimy *et al.* 2023).

ECS can be severe and constitutes an endocrine emergency as life-threatening sequelae of hypercortisolaemia, including hypokalaemia, hypertension, hyperglycaemia, immunosuppression, neurocognitive dysfunction and a procoagulative state, which require urgent treatment alongside cortisol-lowering strategies (Nieman *et al.* 2015, Young *et al.* 2020). The cornerstone of medical therapy for ECS involves the blockade of adrenal cortisol synthesis with agents including metyrapone, osilodrostat, mitotane and ketoconazole or glucocorticoid receptor antagonism with mifepristone. Depending on the agent used for medical control and the underlying disease biology, between approximately 18 and 40% of patients will have ECS that is refractory to medical therapy and requires definitive management – which has traditionally involved surgical resection of the culprit lesion or bilateral adrenalectomy (BA) (Valassi *et al.* 2012, Landry *et al.* 2022, Dormoy *et al.* 2023). NEN resection is generally only considered for solitary lesions – most often a localised bronchial, pancreatic or thymic NET – while patients with metastatic disease require systemic oncologic therapy (Nieman *et al.* 2015, Davi *et al.* 2017, Young *et al.* 2020, Landry *et al.* 2022). BA, usually after a short period of medical therapy, is considered for the control of ECS in patients with non-resectable or metastatic disease, but this leads to lifelong glucocorticoid and mineralocorticoid replacement with the risk of Addisonian crisis. Surgery also carries high peri- and post-operative risks, including complications due to immunosuppression-related sepsis, impaired wound healing, coagulopathy and myopathy associated with ECS (Landry *et al.* 2022).

Peptide receptor radionuclide therapy (PRRT), the delivery of DNA-damaging radiation to cancer cells via targeted radiopharmaceuticals, is an attractive option for patients with ECS caused by functional NEN with high somatostatin receptor (SSTR) expression. PRRT is effective for oncologic control in metastatic NEN (Strosberg *et al.* 2021, Mitjavila *et al.* 2023, Singh *et al.* 2024), but has long been recognised to reduce hormone secretion even in advance of tumour control (Krenning *et al.* 1994). For high-grade NEN (e.g. G3 GEPNET or NEC), there is biological plausibility and emerging clinical evidence for added cytoreductive efficacy using a combination of PRRT with chemotherapy, though this needs to be balanced with the risk of myelotoxicity (Kesavan *et al.* 2021, Nicolini *et al.* 2021, Pavlakis *et al.* 2022). There is established evidence that PRRT treatment of functional NEN leads to a reduction in hormone levels and improvement of clinical syndromes, including carcinoid syndrome, hypoglycaemia from insulinoma and hypertension from phaeochromocytoma/paraganglioma (Kong *et al.* 2017, Zandee *et al.* 2021, Friebe *et al.* 2023). Previous small case series have reported success with PRRT for the management of ECS (Davì *et al.* 2008, Makis *et al.* 2016, Davi *et al.* 2017, Zhang *et al.* 2020). One multicentre retrospective review reported 10/13 patients treated with PRRT had >50% reduction in 24-h urine-free cortisol levels following treatment; however, progression-free survival and overall survival were not described for these patients (Davi *et al.* 2017). Radiosensitivity of ACTH-secreting pancreatic NETs and lengthy durability of hormone and cytologic response to PRRT has been suggested in imaging case reports (Makis *et al.* 2016, Zhang *et al.* 2020); however, detailed clinical, biochemical and imaging follow-up data are lacking. We report here our series of seven consecutive patients who received PRRT in the treatment of ECS, with the aim to evaluate the onset and magnitude of treatment efficacy for cortisol secretion and tumour control, and to provide insight into this unique subtype of functional NEN.

## Materials and methods

### Clinical cohort

Patients with a diagnosis of ECS due to NEN who were referred for consideration of treatment with PRRT at the Peter MacCallum Cancer Centre between 2006 and 2023 were retrospectively identified from electronic medical records and imaging departmental databases. Clinical data collected included patient demographics, tumour histopathological details, haematology and biochemistry results, molecular imaging phenotype (FDG PET/CT, SSTR PET/CT or SPECT/CT for patients treated before 2009), details of cortisol-lowering therapy (CLT) or oncologic therapy prior to PRRT and progression-free survival and overall survival.

This study was approved by The Peter MacCallum Cancer Centre Institutional Ethics Committee QA/79960/PMCC. All patients provided written informed consent to undergo treatment and follow-up.

### Confirmation of ECS diagnosis

An ECS diagnosis was confirmed by endocrinologist assessment at the time of presentation. The diagnosis of ECS was made on the basis of clinical features and biochemical evidence of ACTH-dependent hypercortisolaemia as per national guidelines (Chiang *et al.* 2021). Further dynamic testing (e.g. dexamethasone suppression testing (DST), inferior petrosal sinus sampling or CRH testing) to confirm the ectopic ACTH source was not undertaken in all patients for the following reasons: i) all patients had a known diagnosis of NEN at the time of referral to our centre with ACTH-dependent hypercortisolaemia (Ragnarsson *et al.* 2024) and ii) patients presented with severe ECS manifestations such as critical, intractable hypokalaemia andpsychosis, necessitating urgent institution of adrenal steroid hormone blockade.

### Patient selection for PRRT

Eligibility for PRRT included SSTR-tracer intensity greater than liver on tomographic imaging (i.e. modified Krenning score >2) at all visualised disease sites above the spatial resolution of the imaging technique used (primarily [^68^Ga]Ga-DOTA-octreotate (Ga-Tate) PET/CT or [^111^In]In-pentreotide SPECT/CT prior to 2009). Exclusion criteria for PRRT generally include low SSTR-expression (uptake less than background liver activity) at measurable sites of disease; discordant FDG-avid, SSTR-low lesions, hypoalbuminaemia (<25 g/L), glomerular filtration rate <30 mL/min, thrombocytopaenia (<50 × 10^9^/L), pancytopaenia, ECOG performance status >3, expected survival <3 months or confirmed pregnancy (Hicks *et al.* 2017).

All patients were treated on compassionate grounds under the Australian Government Special Access Scheme, which allows the use of experimental therapies that have demonstrated efficacy in other studies to treat patients with life-threatening diseases.

### Treatment regimen

[^177^Lu]Lu-DOTA-octreotate (Lu-Tate) was the predominant radionuclide therapy administered. One patient with bulky disease (lesions > 4 cm in diameter) received two cycles of [^90^Y]Y-DOTA-octreotate (Y-Tate), followed by two cycles of Lu-Tate. One patient received [^111^In]In-octreotide (In-Tide) before continuing initial therapy with three cycles of Lu-TATE.

Lu-Tate radiolabelling was performed under the institutional protocol, with ^177^Lu labelled to octreotate through chelation to a DOTA molecule by our radiopharmaceutical scientist team. Each cycle of Lu-Tate was administered with a premedication protocol involving antiemetics (ondansetron or granisetron), as well as a renoprotective amino acid infusion (25 g lysine and 25 g arginine in 1 L normal saline), commencing 30 min prior to PRRT and continuing for 3 h thereafter at each cycle. For patients with impaired renal function or receiving Y-Tate, this infusion was continued for 4 h. The usual premedication practice of giving dexamethasone was modified by withholding dexamethasone in 5/7 patients due to excess cortisol secretion. Dexamethasone was administered, however, to one patient with a view to alleviating hepatic capsular inflammation (patient 1, who had previous ECS control with BA) and to another who was at that stage not recognised to have ECS (patient 5). The initial treatment generally comprised four cycles of Lu-Tate given 6–10 weeks apart with typical administered activity of 8 GBq. Variations to this standard administered activity included an increase in patients with a large disease burden (broadly based on the presence of >20 lesions or individual lesions >5 cm), and a reduction in activity for patients with significantly impaired renal function (GFR <60 mL/min), or haematological toxicity from prior cycles of PRRT.

In our department, radiosensitising chemotherapy in addition to PRRT is considered for patients with histology or imaging suggestive of high-grade disease, and who do not have significant contraindications. When given, radiosensitising chemotherapy is added from PRRT cycle 2 onwards and considered for subsequent retreatment cycles. The regimen has changed over the review period. Early patients received 5-fluorouracil (5-FU) 200 mg/m^2^ daily, starting 2 days prior to PRRT for 2 weeks in total. The subsequent institutional protocol included the use of oral capecitabine (825 mg/m^2^ bd commencing 2 days prior to PRRT for 2 weeks) or CAPTEM (capecitabine 750 mg/m^2^ oral bd D1–14 commencing 9 days prior to PRRT, with temozolomide 100 mg/m^2^ bd D10–14 commencing on the day of PRRT for 5 days).

Patients received PRRT retreatment when indicated if the disease retained sufficient SSTR expression and if there was a favourable response to initial PRRT. Earlier, patients were typically prescribed one to two cycles of maintenance PRRT approximately each year after initial therapy. More recently, retreatment has only been considered for patients upon disease progression following an initial response to PRRT.

### Follow-up

Patients were clinically reviewed before and after each cycle of PRRT, and typically at 1–3 month intervals after the last cycle of treatment. FDG and SSTR PET/CT were repeated at approximately 3–6 months following the completion of therapy and compared to baseline. The timing of subsequent follow-up PET/CT was guided by the individual patient’s treating clinician and disease characteristics.

### ECS response

ECS response to PRRT was assessed as a composite of i) change in medical CLT, ii) ACTH levels and iii) cortisol levels. Where applicable, the time to cessation of CLT post cycle 1 of PRRT was measured. ECS remission was defined biochemically as the normalisation of ACTH, hypokalaemia and cortisol despite CLT or potassium supplement cessation, as well as documented resolution of clinical features of Cushing’s syndrome. ECS persistence is defined as the inability to wean CLT due to rebound hypercortisolaemia, hypokalaemia or clinical Cushing’s syndrome. ECS response was not assessable in patients in whom CLT was continued despite clinical, ACTH, anatomical or functional imaging response.

### Molecular imaging response

Molecular imaging response is reported as the response on PET/CT at the first follow-up after PRRT cycle 1 (PRRT-C1). The best observed molecular imaging response is also reported at any time point after PRRT-C1. Ga-Tate PET/CT (or [^111^In]In-pentetreotide SPECT/CT) scan response was defined as complete SSTR response: disappearance of all tracer-avid lesions (or) uptake indistinguishable or less than background physiological uptake if residual anatomical abnormality; partial SSTR response: reduction in the intensity of uptake by one modified Krenning score in at least one tumour site associated with a decrease/stable size on CT (if measurable) or PET (if non-measurable on CT) (or) reduction in the size of tracer-avid lesions (if measurable) regardless of the intensity of uptake; stable SSTR disease: no partial response or progressive disease and progressive SSTR disease: development of new tracer-avid lesions (or) an increase in the size of the tracer-avid lesions on CT (if measurable) or on PET (if non-measurable on CT) regardless of uptake.

Metabolic response was assessed on FDG PET/CT according to the semiquantitative Hicks criteria (Hicks 2005). FDG lesion avidity was defined as the SUVmax of a 1 cm^3^ region of interest over the most avid part of the disease; SUVmax < 5 is ‘mild’, 5–10 is ‘moderate’ and >10 is ‘intense’ avidity (Hofman & Hicks 2016).

### CT response

Stable, partial response, complete response or progression was defined by Response Evaluation Criteria in Solid Tumours (RECIST criteria 1.1). When available, contrast-enhanced CT images were directly compared. Otherwise, non-enhanced CT from PET (or SPECT) components of the study were assessed using scintigraphic uptake to identify the dominant lesions.

### Safety

All haematological and renal toxicities occurring from the time of PRRT administration were recorded and defined according to the Common Terminology Criteria for Adverse Events version 5.0. A ‘flare’ of ECS was defined as documented worsening of the ECS clinical syndrome or admission to hospital for symptom management (e.g. psychiatric disturbance and myopathy), hypokalaemia or fluid balance in the 2 weeks following PRRT administration.

### Statistics

Patient characteristics are described using the median and range for continuous variables. Percentage and count are used for categorical variables. Progression-free survival was defined using clinical (ECS symptom recurrence), imaging (RECIST 1.1 and molecular imaging response as defined above) and biochemical criteria (recurrence of biochemical ECS) after the date of PRRT-C1. Overall survival is defined as the time to death after the date of PRRT-C1. All analyses were performed using R Statistical Software (v4.2.2; R Core Team 2022).

## Results

### Patient demographics

Over the 17-year study period, 24 patients with a diagnosis of ECS had imaging evaluation at our centre (duodenal NET *n* = 1; medullary thyroid cancer (MTC) *n* = 1; pancreatic NET *n* = 8; NEC unknown primary *n* = 2; NET unknown primary *n* = 1; lung NET *n* = 5; lung NEC *n* = 2; rectal NEC *n* = 1; rectal NET *n* = 1 and thymic NET *n* = 2). Of these, 12 patients were unsuitable for PRRT due to metastatic disease with either low SSTR expression (*n* = 10) or discordant FDG-avid non-SSTR-expressing sites of disease suitable for systemic chemotherapy (*n* = 2). A further five patients (four with lung NET and one with MTC) had localised disease suitable for locoregional therapies.

Seven patients with ECS due to NEN (median age: 54 years, range: 17–75; 6 female) underwent PRRT. Five patients had a histological diagnosis of pancreatic NET (WHO Grade 1, *n* = 1; Grade 2, *n* = 3; Grade 3, *n* = 1), one had rectal NET (WHO Grade 1) and one had rectal NEC. Gastrin was the most common co-secreted hormone in addition to ACTH and was documented in 3 of 7 patients. See Table 1 for details of patient characteristics and disease overview, including details of NEN histology, oncologic therapy received prior to PRRT and survival. See supplementary data (see section on supplementary materials given at the end of this article) file for individual patient vignettes.

**Table 1:** Patient clinicopathological demographics, ECS response and survival overview.

Patient	Age/sex	Primary site	Grade (Ki-67%)	Non-ECS hormone cosecretion	Systemic therapy prior to PRRT for NET control	Treatment prior to PRRT for ECS control (see Table 3 for further details)	Initial ECS PRRT no. cycles/treatment regimen (GBq)	ECS response	PFS (months)	Duration of ECS response (months)	Overall survival (months)
1	17F	Pancreas	G1 (ND)	Gastrin and VIP	Imatinib	Bilateral adrenalectomy	1/ ^111^InTide +5FU (7.4) 3/ ^177^LuTate + 5FU (22.8)	Remission (normal ACTH)	208	208	208^b^
2	25F	Pancreas	G3 (50)	None	None	Metyrapone	3/ ^177^LuTate +cap (29.8)	Remission	27	39	39^b^
3	53F	Pancreas	G2 (15)	Gastrin	Lanreotide	Metyrapone	1/ ^90^Y-Tate (4.2) 3/ ^177^LuTate+5FU (21.2)	Remission	46	118	118^c^
4	54F	Pancreas	G3 (50)	None	Carbo/Etop Lanreotide	Metyrapone	2/ ^177^LuTate +cap (16.0)	Remission (transient)	12	12^a^	28^b^
5	58F	Pancreas	G2 (15)	Gastrin	Lu-Tate 32.7GBq	Ketoconazole / metyrapone	2/ ^177^LuTate^#^ (16.3)	Persistence	3	NA	12^b^
6	60F	Rectum	G1 (2)	None	Lanreotide	Metyrapone	4/ ^177^LuTate +cap (34.4)	Not assessable (Remained on CLT, biochemistry normal)	23	23	23^b^
7	75M	Rectum	NEC (>95)	None	Carbo/etop FOLFIRI	Ketoconazole	2/ ^177^LuTate (16.1)	Persistence	3	NA	4^c^

^#^Does not include prior induction PRRT (^177^LuTate 4 cycles/32.7 GBq) 14 months prior to development of ECS; ^a^Underwent elective bilateral adrenalectomy at 18 months; ^b^Alive at time of reporting; ^c^Deceased.

5FU, 5-flurouracil; ^90^Y-Tate, [^90^Y]Y-DOTA-octreotate; ^111^InTide, [^111^In]In-octreotide; ^177^LuTate, [^177^Lu]Lu-DOTA-octreotate; cap, capecitabine; carbo, carboplatin; etop, etoposide; FOLFIRI, folinic acid/fluorouracil/irinotecan; NA, not applicable; ND, not done; NEC neuroendocrine carcinoma; VIP, vasoactive intestinal peptide.

### Baseline imaging phenotype

**Figure 1:**
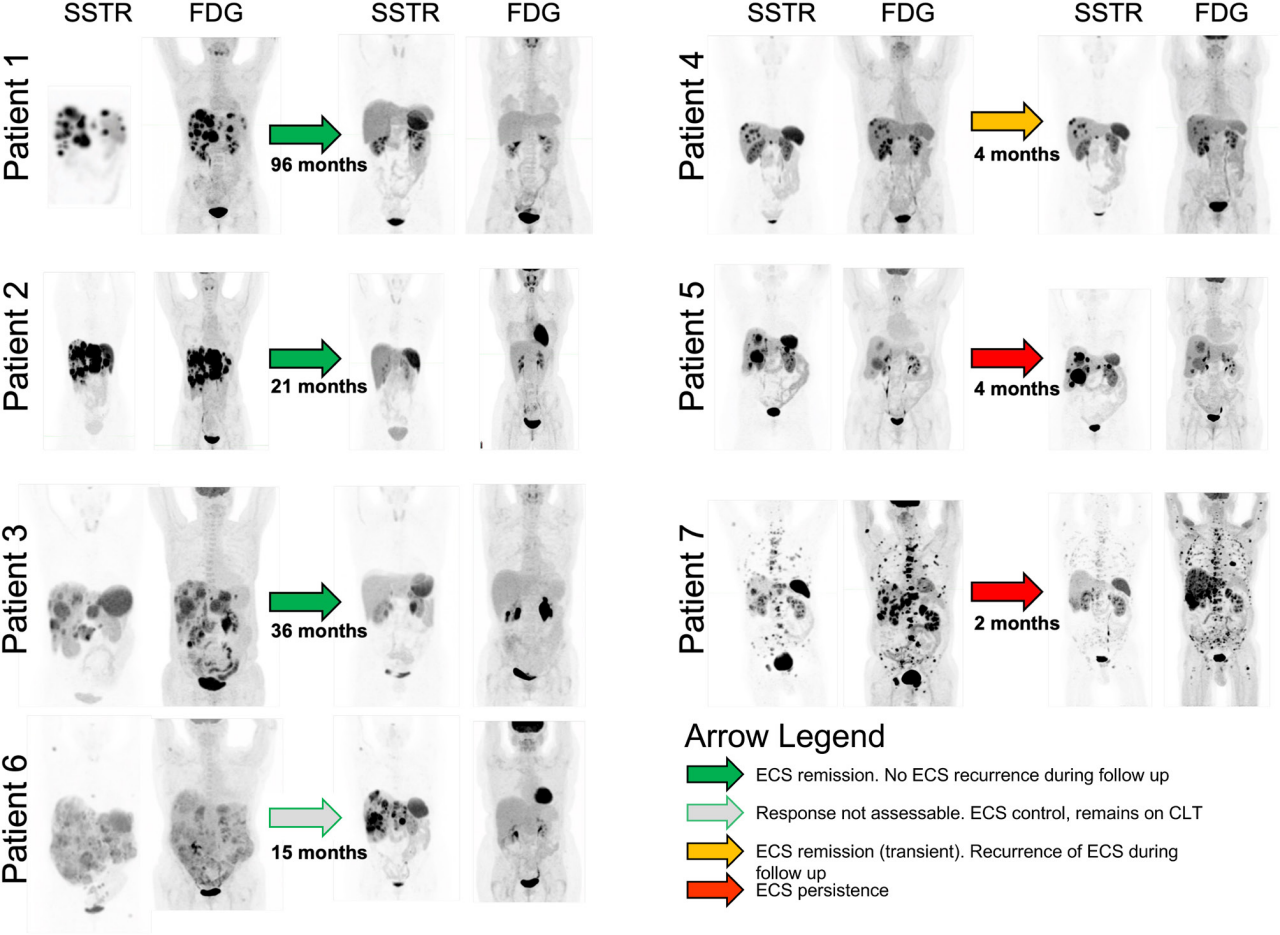
Best imaging response after PRRT for ECS management. SSTR and FDG maximum intensity projections (MIPs) are shown at baseline (immediately prior to PRRT) to the left of the arrow. The SSTR and FDG MIPs to the right of the arrow demonstrate the best imaging response with time to the best response detailed below the arrow for each patient. The arrow colour signifies the biochemical response per patient. CLT, cortisol lowering therapy; ECS, ectopic Cushing’s syndrome; FDG, ^18^Fluorodexoyglucose; SSTR, somatostatin receptor.

As required for allocation to treatment with PRRT, all patients had modified Krenning 3 or higher uptake of tracer on pretherapy SSTR imaging (Table 2 and Fig. 1). All patients had metastatic disease. Metastatic disease distribution was predominantly locoregional nodal and hepatic, with one patient having bony metastatic disease. All patients had moderate to intense FDG uptake on pretherapy assessment indicative of hypermetabolic disease. Interestingly, this included two patients (patients 1 and 6) who had prior biopsies suggestive of low-grade disease. FDG uptake was concordant in all patients except for patient 7, who had discordant sites of FDG-avid, SSTR-negative disease as described below.

**Table 2:** Details of pre-PRRT imaging phenotype, PRRT regimen and imaging response.

Patient	Pre-PRRT FDG avidity	Pre-PRRT SSTR imaging phenotype (modified Krenning score)	Structural response at first follow-up (RECIST)	FDG PET/CT response at first follow-up post induction	SSTR PET/CT response at first follow-up post induction	Best FDG PET/CT response (months post PRRT)	Best SSTR PET/CT response (months post PRRT)	ECS Response	Total number of PRRT cycles / activity administered (GBq)
1	Intense	4	PR	PMR	PR	CMR (45)	CR (96)	Remission	^111^InTide 1/7.4 ^177^LuTate 8/62.7
2	Intense	4	PR	PMR	PR	CMR (21)	CR (21)	Remission	^177^LuTate 5/48.0
3	Intense	4	PR	PMR	PR	CMR (36)	CR (36)	Remission	^90^Y-Tate 2/6.2 ^177^LuTate 13/88.1
4	Moderate	4	PR	PMR	SD	PMR (4)	SD (4)	Remission (transient)	^177^LuTate 2/16.0
5	Moderate	4	SD	PD	SD	PD	SD	Persistence	^177^LuTate 2/16.3*
6	Moderate	4	PR	PMR	PR	CMR (15)	PR (10)	Not assessable (Remained on CLT, biochemistry normal)	^177^LuTate 4/34.4
7	Intense	3	Not assessable	PD	PD	PD	PD	Persistence	^177^LuTate 2/16.1

*Does not include prior induction PRRT (^177^LuTate 4 cycles/32.7 GBq) 14 months prior to development of ECS.

^90^Y-Tate, [^90^Y]Y-DOTA-octreotate; ^111^InTide, [^111^In]In-octreotide; ^177^LuTate, [^177^Lu]Lu-DOTA-octreotate; CLT, cortisol lowering therapy; CMR, complete metabolic response; CR, complete response; PD, progressive disease; PMR, partial metabolic response; PR, progressive response; SD, stable disease.

### ECS diagnosis and pre-PRRT therapy

ECS was present at NEN diagnosis in patients 2, 3, 4, 6 and 7 (see Table 3 for a per-patient breakdown of biochemistry and management). These patients had pre-PRRT biochemistry demonstrating ACTH-dependent hypercortisolism managed with metyrapone (3/5), ketoconazole (1/5) and dual metyrapone/ketoconazole therapy (1/5). Of note, patient 4 had subclinical ECS, which manifested after PRRT-C1 when she was admitted 15 days post treatment with hypokalaemia (potassium 2.9 mmol/L, RR 3.5–4/5 mmol/L) and hypertension due to a flare of ACTH release. The patient reported peripheral oedema and weight gain beginning within 7 days post-C1 PRRT. 08:00 h cortisol was unsuppressed after 1 mg DST at 1444 nmol/L (RR 185–624 nmol/L). Cortisol remained unsuppressed following an 8 mg DST with 08:00 h cortisol measuring 1477 nmol/L (RR 185–624 nmol/L) and ACTH measuring 71.6 pmol/L (<20 pmol/L). A total daily dose (TDD) of 750 mg of metyrapone was commenced.

**Table 3:** Clinical, biochemical and management details of ECS before, during and after PRRT. ACTH results reported as pmol/L as per conversion factor (22) with an upper limit of normal of 20 pmol/L. All baseline measurements were taken prior to commencement of CLT. In patients with block-and-replace CLT and corticosteroid, plasma cortisol measurements were taken prior to the morning dose of corticosteroid. Months reported are months after date of PRRT-C1.

Patient	ECS diagnosis and management prior to PRRT						Flare with PRRT-C1		ECS status post PRRT-C1			ECS status at NEN progression		
	Time from NEN diagnosis to ECS onset	Clinical features	Potassium (RR 3.5–4.5 mmol/L)	ACTH pmol/L	Cortisol P nmol/L U nmol/d	Management	Features	Management	CLT status (months)	ACTH pmol/L (months)	Cortisol P nmol/L U nmol/d (months)	ACTH pmol/L (months)	Cortisol P nmol/L U nmol/d (months)	Management
1	2 years	Not documented	Unknown	98.9*	P 2647*	Bilateral adrenalectomy (urgent)	NA	NA	NA	19.7 (4.5)	NA	NA	NA	NA
2	At NET diagnosis	Amenorrhoea Hirsutism Weight gain	3.1	40.0	P 2675	MET 750 mg Spiro 25 mg	K+ 2.6	IV K MET 1.5g	Ceased (8.25)	4.3 (1.5)	P 128 (1.5)	1.6 (27)	P 248 (27)	NA
3	At NET diagnosis	Peripheral oedema	2.1	96.9	U 49,486	MET 2.25g	K+ 2.4	IV K MET 3g + dex (block/replace)	Ceased (7)	2.0 (4)	<82 (4)	7.2 (46)	P 128 (46)	NA
4	After PRRT-C1	Subclinical until PRRT	2.9^	59.3^	U 16,237^	NA	Peripheral oedema K+ 2.9	IV K MET 750 mg Spiro 50 mg	Ceased (3.75)	12.7 (3.25)	P 182 (3.25)	56.7 (12)	U 5284 (12)	MET 750 mg followed by bilateral adrenalectomy (elective)
5	1.5 years	Psychosis	1.9	131.6	U 1650	MET 750 mg KET 400 mg	Psychosis proximal myopathy ACTH 351.6 U 2102	MET 4g KET 400 mg	MET 1.5g KET ceased (12)	123 (12)	U <7 (12)	290 (3)^#^	U 695 (3)^#^	NA
6	At NET diagnosis	Not documented	1.9	34.8	U 3551	MET 3.75 g + hydrocortisone (block/replace) Spiro 100 mg	NA	NA	MET 1.5g + hydrocortisone (block and replace)	6.5 (6)	P 313 (6)	NA	NA	NA
7	At NET diagnosis	Proximal myopathy	2.2	52.0	P 2584	KET 200 mg Spiro 200 mg	Delirium hyperglycaemia K+ <3.5	IV K (up to 150 mmol/day required)	KET 600 mg	69.8 (2)	P 9089 (2)	69.8 (2)	P 9089 (2)	NA

*Prior to urgent bilateral adrenalectomy; ^1 diagnosed at admission 15 days post PRRT-C1; ^#^2 structural and molecular imaging progression post PRRT C1.

ACTH, adrenocorticotrophic hormone; CLT, cortisol lowering therapy; ECS, ectopic Cushing’s syndrome; IV K, intravenous potassium replacement; K+, potassium (RR 3.5-4.5 mmol/L); KET, ketoconazole total daily dose; MET, metyrapone total daily dose; NA, not applicable; NEN, neuroendocrine neoplasm; P, 08:00 h plasma cortisol; PRRT, peptide receptor radionuclide therapy; PRRT-C1, peptide receptor radionuclide therapy cycle 1; Spiro, spirolactone; U, 24-h urinary-free cortisol.

Two patients developed ACTH secretion and ECS later in their disease course. Patient 1 developed ECS 2 years after the diagnosis of pancreatic NET (grade 1, co-secretory gastrinoma/VIPoma). Patient 1 presented with acute, severe ECS necessitating urgent BA and was on steroid replacement at the time of PRRT. Patient 5 developed ECS 1.5 years after the initial diagnosis of pancreatic NET (grade 2, gastrinoma) with new psychosis and potassium of 1.9 mmol/L (RR 3.5–4.5 mmol/L), necessitating hospital admission. Cortisol was unsuppressed following a 1 mg DST with 08:00 h cortisol measured at 1867 nmol/L (RR 100–540 nmol/L). Cortisol remained unsuppressed after an 8 mg DST: on the morning of the 8 mg DST, the cortisol was 1200 nmol/L (RR 100–540 nmol/L); following 8 mg of dexamethasone administered intravenously at 23:00 h, the 08:00 h cortisol was unsuppressed at 1198 nmol/L (RR 100–540 nmol/L). Metyrapone TDD 750 mg plus ketoconazole TDD 400 mg was commenced.

### Pre-PRRT oncologic therapy

Two patients underwent surgical resection of primary or metastatic disease prior to PRRT. One patient had received imatinib. Three patients received lanreotide, which was stopped 4–6 weeks prior to PRRT. Two patients received carboplatin/etoposide chemotherapy and one of these patients (patient 7, rectal NEC) also received FOLFIRI prior to PRRT. Patient 5 had PRRT (Lu-Tate 32.7 GBq) for gastrin-secretory pancreatic NET G2, prior to the development of ECS and had a partial SSTR response (PFS = 11.5 months). See Table 1 for details of previously received systemic therapy.

### PRRT details

All patients received LuTate therapy over a median of four cycles (range: 2–15). A median of 34.4 GBq (range: 16.0–88.1) was administered. Five patients had concomitant radiosensitising chemotherapy for at least one cycle. One patient had In-Tide (7.4 GBq), and one patient had two cycles of Y-Tate (total of 6.2 GBq) as part of the initial therapy. See Table 1 for a per-patient breakdown of the therapy received. Three patients (patients 1, 2 and 3) had retreatment PRRT after initial therapy (see Table 2 for details of total PRRT exposure). Reasons for retreatment were consolidation of initial treatment response (patient 1) and disease progression following an initial response (patients 2 and 3; see below for details of progression-free survival).

### Response to PRRT

#### Ectopic Cushing’s syndrome

**Figure 2:**
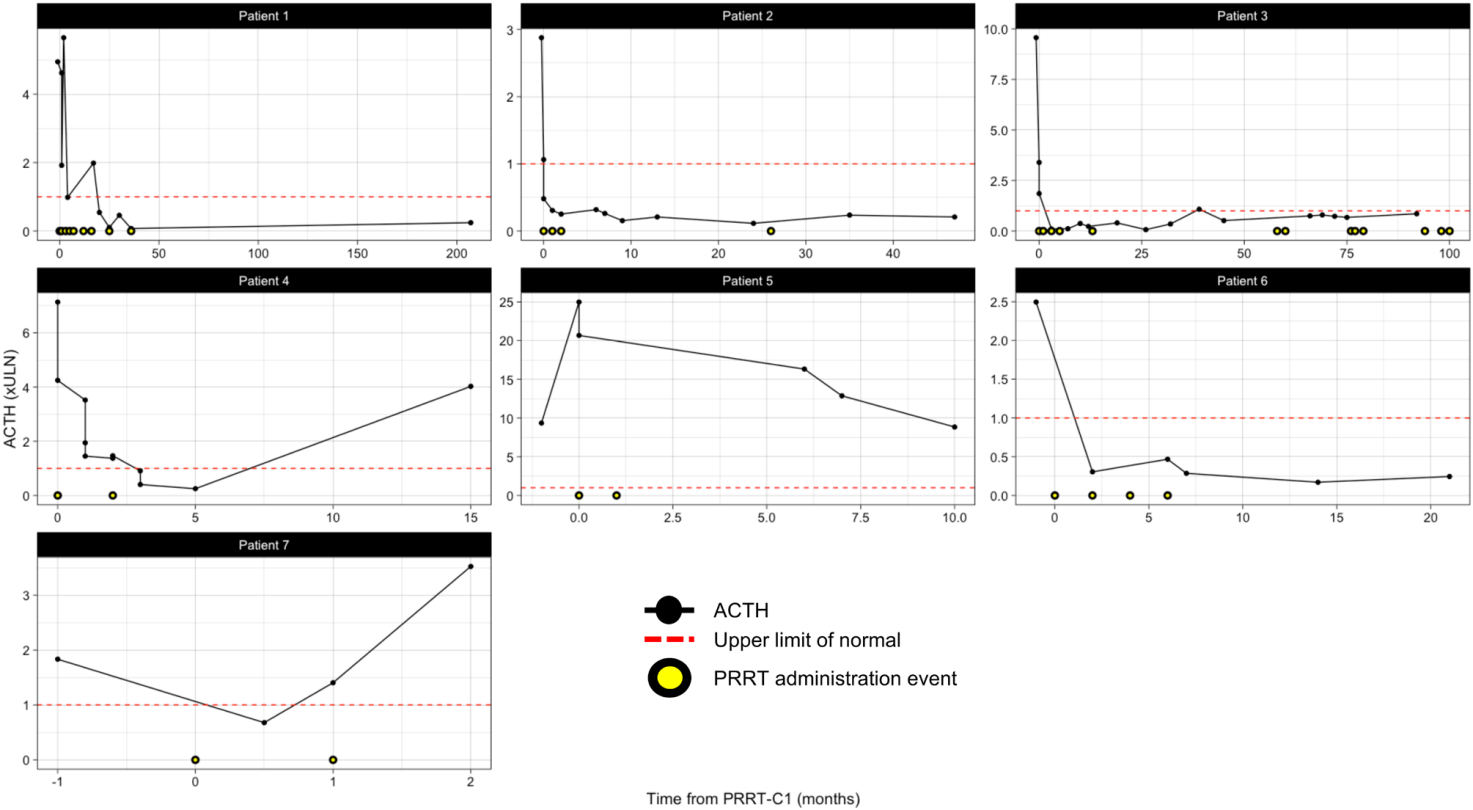
Biochemical response to PRRT. ACTH levels were measured throughout the ECS and NEN disease course for each patient in relation to the date of PRRT cycle 1 and subsequent cycles.

Following initial PRRT, five patients had normalisation of ACTH and cortisol as biomarkers for disease activity (see Table 3 and Fig. 2 for details). The time taken for the resolution of biochemical ECS following the first cycle of PRRT ranged between 6 weeks and 6 months. Furthermore, three of these patients were able to stop CLT after 3.8–7 months, with one patient continuing at the discretion of the treating physician (Table 3). The duration of ECS response ranged from 12 to 208 months. Only one responder, patient 4, had a recurrence of ECS at the time of disease progression, 12 months post PRRT, requiring reinstitution of metyrapone TDD 750 mg and proceeded to elective BA. One patient remains in ECS remission at 208 months.

Two patients did not have complete resolution of ECS post therapy. Patient 5 had previous treatment with four cycles of LuTate (32.7 GBq, one cycle with radiosensitising chemotherapy, partial response with structural progression after 11.5 months) and developed new ACTH hormone secretion and ECS detected 14 months after initial PRRT. PRRT retreatment was complicated by a biochemical and clinical flare of Cushing’s syndrome (see below section on safety and Table 3) requiring escalation of metyrapone dose to 4 g/d. Elevated ACTH (123 pmol/L, <20 pmol/L) and requirement for CLT persisted 12 months post cycle 1, indicating ongoing hormone production by the patient’s NET. Plasma and 24-h urinary free cortisol; however, normalised. Ketoconazole was stopped, and medical control was achieved on a substantially reduced dose of metyrapone (from 4 g/d to 1.5 g/d), suggesting a partial biochemical response to PRRT. The second patient who did not have ECS remission, patient 7, had a diagnosis of rectal NEC (Ki67 95%) and had been heavily pre-treated with carboplatin/etoposide (six cycles) and FOLFIRI (six cycles). This patient experienced regression of ECS and was able to stop CLT after both lines of chemotherapy. ECS recurred with each episode of disease progression. At the time of LuTate (without radiosensitisation), this patient was ECOG 3, and had sites of discordant FDG-avid, SSTR-negative disease, but the patient elected to undergo treatment for palliative purposes given limited other options. This patient ultimately progressed with FDG-avid disease and was deceased within 4 weeks of cycle 2 of PRRT.

#### Imaging response

On initial follow-up at 3–6 months post-PRRT, five of seven patients had a partial response and one had stable disease as per RECIST 1.1 criteria. One patient (a non-responder, patient 7) did not have measurable structural disease but had progressive, predominantly osseous, disease on FDG and SSTR PET/CT 1 month after PRRT (Table 2).

The best molecular imaging response was remarkable for four patients having a complete metabolic response on FDG/PET CT (15, 21, 36 and 45 months post-PRRT), three of whom had complete resolution of SSTR-expressing disease (Fig. 1). The metabolic response on FDG PET/CT preceded the SSTR response. These patients also had complete resolution of ECS, which did not recur (follow-up 23–208 months post-PRRT, see Table 1). Patient 1 continues to have no detectable disease on molecular imaging more than 14 years post-PRRT. Patient 6 has achieved a CMR on FDG PET/CT and has a partial response on SSTR PET/CT with biochemical ECS remission and follow-up ongoing. One patient (patient 4) had a PMR on FDG PET/CT and stable SSTR-expressing disease. This patient, as previously described, had initial improvement of ECS, which recurred at the time of imaging progression. Two patients had progression on FDG PET/CT and had persistence of ECS post-PRRT.

#### Survival outcomes

Of the five patients who demonstrated imaging and ECS response, progression-free survival ranged from 12 to 208 months. Patient 6 and patient 1 have ongoing imaging and biochemical response (at 23 and 208 months, respectively, post-PRRT-C1), while the other three patients progressed after 12, 27 and 46 months respectively. Overall survival ranged from 3.8 to 208 months. The two deaths in the cohort were attributable to metastatic disease progression and occurred at 3.8 months (in the context of poor prognostic factors, including NEC Ki67 95%, FDG-avid and high burden disease) and 118 months post PRRT.

#### Safety

Five patients had a clinical flare of ECS documented within 7 days of PRRT (Tables 3 and 4). Two of these patients required hospitalisation for the management of severe hypokalaemia, hypertension and, in one, psychiatric disturbance. All patients received ward-based care, with no patient requiring escalation to an intensive care setting.

**Table 4:** Safety data. Details of ECS flare post-PRRT and haematological toxicity. All haematological toxicity was transient.

Patient	Flare of ECS with PRRT (cycle)	Other PRRT toxicity
1	NA (prior bilateral adrenalectomy)	G1 anaemia
2	Yes (C1)	G1 thrombocytopaenia
3	Yes (C1)	NA
4	Yes (C1)	G1 anaemia G3 thrombocytopaenia
5	Yes (C1)	G1 Lymphopaenia G1 thrombocytopaenia
6	No	NA
7	Yes (C1)	NA

Haematological toxicity of one or more lineages occurred in four patients and was transient. This included grade 1 anaemia (*n* = 2), grade 1 lymphopaenia (*n* = 1), grade 1 thrombocytopaenia (*n* = 1) and G3 thrombocytopaenia (*n* = 1) (Table 4). No patient developed treatment-related myeloid neoplasia (t-MN) on long-term follow-up.

## Discussion

ECS is associated with significant morbidity and mortality. In the case of metastatic NEN, patients face the compounding physiologic insults of extensive disease burden and the sequelae of hypercortisolaemia. As such, they are often poor surgical candidates for BA at initial presentation, and stabilisation with medical CLT is necessary, but complete control is reportedly low at <30% (Davi *et al.* 2017). In this case series of patients with metastatic GEP NEN, we showed that PRRT with radiosensitising chemotherapy can be effective for achieving ECS control in patients with high SSTR-expressing disease. A significant proportion of patients (5/7, 71%) achieved normalisation of ACTH as a biomarker of hormone secretion, all within 6 months from PRRT-C1. Three patients were able to stop CLT altogether. Only one patient (patient 4) had a recurrence of ECS on disease progression and was able to safely proceed to elective BA. Others had an overall durable effect (23–208 months).

Importantly, this case series also adds to the growing literature regarding PRRT for oncologic control in high-grade GEPNEN patients (Singh 2024), outside of the grade 1–2 cohorts defined in the seminal NETTER trial (Thang *et al.* 2018, Carlsen *et al.* 2019). Indeed, the patients in our case series included histologically defined G3 NET (2/7) or NEC (*n* = 1) or had imaging characteristics suggestive of metabolically active disease with intense metabolic activity on FDG PET/CT. Both patients with G3 NET (Ki 67 50%) achieved ECS remission and favourable imaging responses post PRRT. The patient with NEC (Ki67 95%), however, progressed rapidly post PRRT, but in the context of poor prognostic factors, including high-grade pathology, high-burden disease and the presence of a baseline discordant molecular imaging phenotype. The failure to control ECS, in this case, suggests that ectopic cortisol may be particularly associated with metabolic reprogramming and altered hormonal synthesis pathways in higher grade NET, as reflected by the association of ECS with small-cell lung cancer. This series highlights the utility of dual tracer FDG and SSTR imaging in selecting patients with NEN who would likely benefit from PRRT. PRRT is unlikely to target all sites of disease with a discordant FDG-positive but SSTR-negative phenotype (Kong & Hicks 2021).

In addition to ECS management with medical therapy and the conventional pathway of invasive BA, we suggest that the management algorithm for ECS should include an evaluation for suitability for PRRT. This should be done in a multidisciplinary setting, and ideally incorporate dual SSTR and FDG PET/CT imaging to characterise disease phenotype to guide suitability for PRRT with consideration for radiosensitising chemotherapy. We propose that PRRT should be given in conjunction with medical CLT and inpatient monitoring at a minimum following cycle 1, given the high risk of ECS flare (5/7 patients in this series).

What is unclear is whether it is safe to delay BA in patients with ECS who are potential surgical candidates. There is currently insufficient data to define biomarkers that predict which patients will have a rapid hormonal response to PRRT (as in patient 2), or otherwise have a delayed response or be refractory. However, BA in patients with uncontrolled Cushing’s and advanced malignancy is often high risk. Sequencing of BA vs PRRT should be considered on a case-by-case basis. PRRT will likely have effects both on ECS hormonal and oncological control and may delay invasive surgical adrenalectomy if PRRT is available for suitable patients in a timely manner. Additionally, it is unclear whether PRRT with or without radiosensitising chemotherapy is superior to chemotherapy or tyrosine kinase therapy alone for ECS control, despite emerging evidence for oncologic benefit and current trials are ongoing, including COMPETE (vs everolimus, NCT03049189), COMPOSE (vs CAPTEM/FOLFOX or everolimus, NCT04919226) and ComPareNET (LuTate vs CAPTEM, NCT05247905) (Baudin *et al.* 2022). We hypothesise that in patients with high somatostatin expression, PRRT will be better tolerated and more effective, but prospective trials are awaited. Due to the rarity of GEPNEN with ECS, randomised controlled trials are unlikely to be feasible, highlighting the importance of case series publication. We and others have highlighted that PRRT is an effective and safe therapy for this indication, as long as caution is exercised regarding monitoring and supportive therapy for ECS flare after cycle 1 and the known reported serious adverse effect of treatment-related myeloid neoplasia is acknowledged (Goncalves *et al.* 2019).

This study has the well-recognised limitations inherent in a retrospective, small case series, with heterogeneous treatment protocols. Reporting our carefully annotated cohort is worthwhile; however, given the rarity of ECS, the requirement for individualised treatment of patients with ECS and NEN, and the limited published experience with the treatment of ECS with PRRT. Additionally, we have only reported outcomes for patients who proceeded to PRRT and not describe the patients who underwent imaging evaluation and did not have modified Krenning 3 or higher SSTR expression. This latter patient group is not suitable for SSTR-targeting PRRT and represents a subset of patients for whom the development of novel theranostic targets or other therapies is imperative.

## Conclusion

Our case series highlights an interesting subset of GEPNEN patients with hormonal secretion (ACTH or CRH), high-grade yet high SSTR-expressing disease who have the potential to respond to PRRT with exquisite hormonal and oncologic sensitivity. It is currently unknown what the underlying driver for this exceptional response is and whether ECS is a biomarker for PRRT sensitivity or an accidental by-product of underlying genomic changes (e.g. hypomethylation, genomic instability or DNA repair deficiency) or high proliferative activity that would render non-ECS-causing NEN similarly sensitive. Further research to understand NEN biology and treatment response will assist with evidence-based, individualised treatment for patients with this heterogeneous disease. In the meantime, we highlight that PRRT may be an effective and safe option for hormone control in patients with ECS due to metastatic GEPNEN with high somatostatin expression, which may delay or avoid BA and provide the dual benefit of peptide and oncologic control. Multidisciplinary consideration of treatment sequencing and management of post-PRRT Cushing’s flare should be embedded in local departmental protocols.

## Supplementary Materials

supplementary Data

## Declaration of interest

The authors declare that there is no conflict of interest that could be perceived as prejudicing the impartiality of the study reported.

## Funding

This work did not receive any specific grant from any funding agency in the public, commercial or not-for-profit sector.

## Data availability statement

Data sharing is not applicable to this article as no new data were created or analysed in this study.

## Acknowledgements

There has been no financial support for this work that could have influenced its outcome. This study was conducted according to the Declaration of Helsinki. This study was registered with The Peter MacCallum Cancer Centre Institutional Ethics Committee (QA/79960/PMCC).
