# Supplementary material for: Peptide receptor radionuclide therapy for ectopic Cushing’s syndrome caused by metastatic neuroendocrine neoplasms
Source: Endocr Oncol. 2024 Nov 20;4(1):e240013. doi: 10.1530/EO-24-0013 (PMC11623253; doi:10.1530/EO-24-0013)
Supplement: supplementary Data [file supplementary_data.pdf]

Peptide receptor radionuclide therapy for ectopic Cushing's Syndrome caused by metastatic neuroendocrine neoplasia

Emma Boehm[1], Terry Hung[1], Tim Akhurst[1], Ramin Alipour[1], Cherie Chiang[2], Rodney J Hicks[3], Michael S Hofman[1], Aravind S Ravi Kumar[1,4], Nirupa Sachithanandan[2], Javad Saghebi[1], Michael Michael[4,5], Grace Kong[1,4]

1. Department of Molecular Imaging and Therapeutic Nuclear Medicine, Peter MacCallum Cancer Centre, Melbourne, Australia
2. Department of Internal Medicine, Endocrinology, Peter MacCallum Cancer Centre, Melbourne, Australia
3. Department of Medicine, St Vincent's Medical School, The University of Melbourne, Melbourne, Australia
4. Sir Peter MacCallum Department of Oncology, University of Melbourne.
5. Department of Medical Oncology, Peter MacCallum Cancer Centre, Melbourne

SUPPLEMENTAL DATA

Case Vignettes

Patient 1 was a 17-year-old female who developed ECS two years after diagnosis of metastatic pancreatic NET (grade unknown, initially co-secretory gastrinoma/VIPoma, treated with pancreatectomy and then imatinib at disease recurrence). Concurrent with disease progression on imatinib patient 1 developed acute, severe ECS necessitating urgent bilateral adrenalectomy at the referring tertiary paediatric hospital. Due to imaging disease progression, PRRT was offered. Immediately prior to PRRT Patient 1 was taking hydrocortisone replacement and ACTH was elevated (98.9pmol/L, ULN <20pmol/L). *PRRT treatment and ECS response*: 7.4GBq of In-Tide with 5FU (cycle 1) followed by 22.8GBq Lu-Tate with 5FU (cycles 2-4) were administered. ACTH was normal at 19.7pmol/L (<20pmol/L) 4.5 months after PRRT-C1. Over the following four years the patient received a further five consolidation cycles of Lu-Tate and ACTH remained within the normal range, last measured at 4.9pmol/L (<20pmol/L) 17 years after first PRRT. *Follow-up imaging response*: Following initial PRRT there was PR on structural imaging, PR on SSTR-imaging and PMR on FDG

PET/CT. *Best molecular imaging response:* Patient 1 achieved a CMR on FDG PET/CT and CR on SSTR-PET/CT 45 months and 96 months, respectively, after PRRT-C1. *Survival outcomes*: Patient 1 has no clinical nor imaging evidence of disease progression, and remains alive and under follow-up at 208 months post PRRT-C1.

Patient 2 was a 25-year-old female diagnosed with metastatic pancreatic NET Grade 3 and ECS after presenting with abdominal pain, 2-months of amenorrhoea, hirsutism and weight gain. At diagnosis patient was normotensive, hypokalemic (K = 3.1mmol/L, RR: 3.5-4.5mmol/L), with ACTH dependent hypercortisolism: ACTH was 40.0 pmol/L (<20pmol/L), 8am cortisol was 2675nmol/L (145-619nmol/L). Immediately prior to PRRT the patient was taking a total daily dose (TDD) of 750mg metyrapone and 25mg of spironolactone. *PRRT treatment and ECS response:* Patient 2 was treated with a total of 29.8GBq of Lu-Tate over 3 cycles, with capecitabine for radiosensitization. Within 24 hours of PRRT-C1 patient 2 developed hypokalaemia (potassium 2.6mmol/L, 3.5-4.5mmol/L) requiring intravenous replacement and metyrapone increase to a TDD of 1.5g, consistent with a treatment-related flare of ECS. ACTH normalised 1.5 months after PRRT-C1 (4.3pmol/L, <20pmol/L). The patient's menstrual cycle returned 2.5 months after PRRT-C1. Metyrapone and spironolactone were weaned and stopped 7-months after initiation of PRRT, indicating ECS remission. Over the following 18 months the patient received a further two retreatment cycles of Lu-Tate. ACTH and 8am cortisol remained within the normal range, including ACTH of 1.6pmol/L (<20pmol/L) and cortisol of 248nmol/L (RR 145-619nmol/L) at time of disease progression on imaging at 27 months post PRRT-C1. *Follow-up imaging response*: Following initial PRRT there was PR on structural imaging, PR on SSTR-imaging and PMR on FDG PET/CT. *Best molecular imaging response:* Patient 2 achieved both a CMR on FDG PET/CT

and CR on SSTR-PET/CT 21 months after cycle PRRT-C1. *Survival outcomes*: Imaging PFS was 27 months. The patient remains alive without recurrence of ECS at the time of last follow-up, 39 months post PRRT-C1.

Patient 3 was a 53-year-old female diagnosed with metastatic pancreatic NET Grade 2 and ECS after presenting with abdominal pain, peripheral oedema and severe hypokalaemia (potassium 2.1mmol/L, 3.5-4.5mmol/L). The ACTH was 96.9pmol/L (<20pmol/L), 24-hour urinary free cortisol was 49,486nmol/d (<350nmol/d). Patient 3 was taking a TDD of 2.25g metyrapone immediately prior to PRRT. *PRRT treatment and ECS response:* Patient 3 was treated with 1 cycle of Y-Tate (4.2GBq) and a total of 21.2GBq of Lu-Tate with 5FU for radiosensitization over 3 cycles. The patient was admitted nine days after PRRT-C1 (Y-Tate) after follow-up biochemistry demonstrated a potassium of 2.4mmol/L (RR 3.5-4.5mmol/L) necessitating intravenous replacement, attributable to a flare of ECS. Metyrapone was increased to a TDD of 3g and dexamethasone replacement was added. Four months after PRRT-C1 ACTH was 2.0 pmol/L (<20pmol/L), and 24-hour urinary free cortisol was <82nmol/d (<350nmol/d), metyrapone was subsequently reduced to a TDD of 1.5g. Seven months after PRRT-C1 ACTH was 2.3pmol/L (<20pmol/L), urinary free cortisol was 70nmol/d (< 350nmol/d), metyrapone was ceased and dexamethasone was subsequently weaned and stopped, indicating ECS remission. Over the following seven years the patient received a further nine re-treatment cycles of PRRT and ACTH remained within the normal range, including at 7.2pmol/L (<20pmol/L) with 24-hour urinary free cortisol of 128 (<350nmol/d) at time of disease progression on imaging at 46 months. The patient remained in ECS remission until death from cancer-related non-hormonal causes. *Follow-up imaging response*: Following initial PRRT there was PR on structural imaging, PR on SSTR-imaging and

PMR on FDG PET/CT. *Best molecular imaging response:* Patient 3 achieved both a CMR on FDG PET/CT and CR on SSTR-PET/CT 36 months after PRRT-C1. *Survival outcomes*: Imaging PFS was 46 months. Cancer-related death occurred 118 months after PRRT-C1.

Patient 4 was a 54-year-old female who had a diagnosis of pancreatic NET Grade 3 and was initially treated with hepatic metastatectomy and carboplatin/etoposide. The patient was referred for PRRT due to imaging progression. Patient 4 had undiagnosed subclinical ECS which become overt after PRRT-C1. *PRRT treatment and ECS response:* Patient 4 received 16.0GBq of Lu-Tate with capcitabine radiosensitization over 2 cycles, having a truncated treatment course due to persisting CTCAE Grade 3 thrombocytopaenia. The patient reported peripheral oedema and weight gain beginning within 7 days post PRRT-C1 and was admitted at day 15 with hypokalaemia (potassium 2.9mmol/L, 3.5-4.5mmol/L), and hypertension (BP 135/85) due to a flare of ACTH release (ACTH 59.3pmol/L, <20pmol/L; 24-hour urinary free cortisol 16,237nmol/d, <305nmol/d). 8am cortisol was unsuppressed after 1mg dexamethasone suppression text (DST) at 1444nmol/L (RR 185 – 624 nmol/L). Cortisol remained unsuppressed following an 8mg DST with 8am cortisol measuring 1477 nmol/L (RR 185 – 624 nmol/L) and ACTH measuring 71.6pmol/L (<20pmol/L). A TDD of 750mg metyrapone plus spironolactone 50mg was instituted to achieve normokalaemia. Six weeks after PRRT-C1 hydrocortisone was added due to development of symptomatic postural hypotension, ACTH was 27.3pmol/L (<20pmol/L) and 8am cortisol 171nmol/L (185-624nmol/L). The ACTH normalised to 12.7pmol/L (<20pmol/L) after 3.25 months and metyrapone was stopped 3.75 months post PRRT-C1 indicating ECS remission. 12 months post PRRT-C1 the patient developed a recurrence of ECS at time of disease progression, with ACTH of 56.7pmol/L (<20pmol/L) and 24-hour urinary free cortisol of 5284nmol/d

(<305nmol/d). Metyrapone TDD of 750mg was re-instituted and the patient proceeded to elective bilateral adrenalectomy. *Follow-up imaging response*: Following initial PRRT there was PR on structural imaging, SD on SSTR-imaging and PMR on FDG PET/CT; this also represents the best imaging response achieved. *Survival outcomes*: Imaging PFS was 12 months. The patient remains alive and under follow-up at 28 months post cycle 1 PRRT.

Patient 5 was a 58-year-old female who had an initial diagnosis of pancreatic NET Grade 2 (gastrinoma) and received initial treatment with 4 cycles of Lu-Tate (32.7 GBq, one cycle with radiosensitizing capecitabine) achieving a partial response on SSTR-imaging. 14 months after cycle 1 of initial PRRT, patient 5 presented with severe hypokalaemia (potassium 1.9mmol/L, 3.5-4.5mmol/L) and psychosis necessitating hospital admission. Molecular imaging progression and ECS were diagnosed: ACTH was 131.6pmol/L (<20pmol/L) and 24-hour urinary free cortisol was 1650nmol/d (<280nmol/d). Cortisol was unsuppressed following a 1mg DST with 8am cortisol measured at 1867 nmol/L (RR 100 – 540nmol/L). Cortisol remained unsuppressed after an 8mg dexamethasone suppression test: On the morning of the 8mg DST the cortisol was 1,200nmol/L (RR 100-540nmol/L); following 8mg of dexamethasone administered intravenously at 11pm the 8am cortisol was unsuppressed at 1,198nmol/L (RR 100 – 540nmol/L). Immediately prior to PRRT the patient was taking a TDD of 750mg metryrapone and 400mg ketoconazole. *PRRT and ECS response:* 16.3 GBq of Lu-Tate was given over two cycles. Cycle 1 of retreatment was complicated by a flare of ECS with worsening psychosis and proximal myopathy associated with ACTH 351.6 pmol/L (<20pmol/L) and 24-hour UFC 2102nmol/d (<280nmol/d) measured four days post treatment. Metyrapone was increased to a TDD of 4g. Elevated ACTH (123pmol/L, ULN <20pmol/L) and requirement for cortisol lowering therapy persists 12 months post PRRT-C1.

Plasma and 24-hour urinary free cortisol, however, normalised and metyrapone was weaned to a TDD of 1.5g with ketoconazole cessation, suggesting a partial biochemical response to PRRT. *Follow-up imaging response:* Following PRRT re-treatment there was SD on structural imaging, SD on SSTR-imaging and progression on FDG PET/CT; this also represents the best imaging response achieved. *Survival outcomes*: PFS was 3 months, and the patient remains alive and under follow-up at the time of reporting, 12 months post PRRT re-treatment.

Patient 6 was a 60-year-old female and was diagnosed with metastatic rectal NET Grade 1 and ECS during a hospital admission for severe hypokalaemia (potassium 1.9mmol/L, 3.5-4.5mmol/L). At diagnosis the ACTH was 34.8pmol/L (<20pmol/L) and the 24-hour urinary free cortisol was 3551nmol/d (<305nmol/d). Prior to PRRT the patient was taking a block-and-replace regimen of metyrapone 3.75g TDD and hydrocortisone, plus spironolactone 100mg daily to maintain normokalaemia. *PRRT treatment and ECS response:* The patient received 34.4GBq of Lu-Tate over 4 cycles with capecitabine radiosensiting chemotherapy. There was no clinical or biochemical ECS flare with PRRT and after five months metyrapone was reduced to a TDD of 3g. Six months after cycle 1 of PRRT the ACTH was 6.5pmol/L (<20pmol/L) and the 8am plasma cortisol was 313nmol/L (185-624nmol/L), and metyrapone was reduced to a TDD of 2.25g. Two months later the ACTH remained normal at 4.1pmol/L (<20pmol/L) and metyrapone was reduced to a TDD of 1.5g with ongoing hydrocortisone replacement. The patient remained on a block-and-replace regimen of metyrapone and hydrocortisone at the discretion of the treating endocrinologist, and ACTH remained normal 21-months after PRRT-C1 at 3.4pmol/L (<20pmol/L). *Follow-up imaging response:* Following PRRT there was PR on structural imaging, PR on SSTR-imaging and PMR on FDG PET/CT. *Best*

molecular imaging response: Patient 6 achieved a CMR on FDG PET/CT and PR on SSTR-PET/CT 15 and 10-months, respectively, after PRRT-C1. *Survival outcomes*: The patient has no clinical nor imaging evidence of disease progression and remains alive and under follow-up at 23 months after PRRT-C1.

Patient 7 was a 75-year-old male who had a diagnosis of rectal NEC (Ki67 95%). The patient had a baseline ACTH of 64.7pmol/L (<20pmol/L) and 24-hour urinary free cortisol of 83,696nmol/d (<305nmol/d). Prior to referral to our centre the patient had been heavily pre-treated with carboplatin/etoposide (6 cycles) and FOLFIRI (6 cycles). This patient experienced regression of ECS and was able to stop CLT after both lines of chemotherapy. ECS recurred with each episode of disease progression and prior to PRRT ACTH was 52pmol/L (ULN <20pmol/L), 8am plasma cortisol was 2584nmol/L (RR 145-615pmol/L) and 24-hour urinary cortisol was not done. This was associated with a potassium of 2.2mmol/L (RR 3.5-4.5mmol/L). Due to proximal myopathy and frailty at the time of PRRT this patient was ECOG 3. The pre-PRRT molecular imaging demonstrated discordant FDG-avid, SSTR-negative disease but the patient elected to undergo treatment for palliative purposes given limited other options. The patient was intolerant of metyrapone and prior to PRRT was taking ketoconazole 200mg daily and spironolactone 200mg daily. *PRRT treatment and ECS response:* The patient received 16.1 GBq of Lu-Tate over two cycles. He was admitted for PRRT-C1 and within 24-hours developed severe hypokalaemia requiring intravenous replacement (up to 150mmol/d), glycaemic instability and delirium attributable to a flare of ECS with treatment. Ketoconazole was increased to a TDD of 600mg. ACTH and plasma cortisol remained elevated at 69.8pmol/L (<20pmol/L) and 9089nmol/L (<485nmol/L) two months after PRRT-C1. *Imaging response* The patient did not have measurable structural

disease but had progressive, predominantly osseous, disease on FDG and SSTR PET/CT at follow-up done early due to clinical deterioration one month after completion of PRRT. *Survival outcomes* The patient died due to disease progression four months after PRRT-C1.
